# A Metabolic Profiling Study of Realgar-Induced Acute Kidney Injury in Mice

**DOI:** 10.3389/fphar.2021.706249

**Published:** 2021-08-23

**Authors:** Sheng Zhang, Chao Li, Tingting Feng, Shuai Cao, Heng Zhou, Limin Li, Qing Hu, Xiuhong Mao, Shen Ji

**Affiliations:** ^1^ Tianjin University of Traditional Chinese Medicine, Tianjin, China; ^2^ NMPA Key Laboratory for Quality Control of Traditional Chinese Medicine, Shanghai Institute for Food and Drug Control, Shanghai, China; ^3^ Shanghai University of Traditional Chinese Medicine, Shanghai, China

**Keywords:** realgar, acute kidney injury, metabolomics, serum, kidney, LC-MS

## Abstract

Realgar has been used as a type of mineral drug that contains arsenic for thousands of years. Previous studies have shown that Realgar-induced acute kidney injury is associated with abnormal metabolism, but the underlying mechanism is poorly understood. The aim of this study is to investigate the metabolic changes in serum and kidney tissues of mice exposed to Realgar by using a metabolomic approach and explore the molecular mechanisms of acute kidney injury induced by Realgar. Forty mice were randomly divided into four groups: Control group, 0.5-, 1.0, and 2.0 g/kg Realgar group. After 1 week, the body weight and kidney weight of the mice were measured. The serum and kidney samples were used for LC-MS spectroscopic metabolic profiling. Principal component analysis (PCA), correlation analysis, and pathway analysis were used to detect the nephrotoxic effects of Realgar. Body weight decreased significantly in the 2.0 g/kg group, and the kidney weight index also showed a dose-dependent increase in Realgar. The PCA score plot showed the serum and kidney tissue metabolic profile of mice exposed to 2.0 g/kg Realgar separated from the control group, while the lower-doses of 0.5 g/kg and 1.0 g/kg Realgar shown a similar view to the Control group. Thirty-three metabolites and seventeen metabolites were screened and identified in the serum and kidney of mice in a dose-dependent manner. respectively. Correlation analysis showed a strong correlation among these metabolites. Amino acid metabolism, lipid metabolism, glutathione metabolism, and purine metabolism pathways were found to be mainly associated with Realgar nephrotoxicity. This work illustrated the metabolic alterations in Realgar-induced nephrotoxic mice through a metabolomic approach.

## Introduction

Arsenic has long been known as a toxic compound and environmental carcinogen, which exposure causes oxidative stress, apoptosis, chromosomal abnormalities, and DNA damage (Zhou and Xi, 2018; Palma-Lara et al., 2020). However, Arsenic has shown amazing therapeutic value in the treatment of hematopoietic malignancies (Zhu et al., 2019). Arsenic trioxide, as an arsenic-containing compound, has shown good remission in the treatment of acute promyelocytic leukemia (APL) (Min et al., 2020). Arsenic trioxide shows great toxic effects and causes serious adverse reactions especially in the case of long-term use (Wang et al., 2020).

Realgar, a traditional Chinese mineral medicine containing more than 90% tetraarsenic tetrasulfide (As_4_S_4_), has been widely used to treat infection, fever, and convulsion (Liu et al., 2018). Recently, Realgar has similar efficacy to arsenic trioxide in the treatment of APL and has fewer side effects (Wu et al., 2011). However, arsenic contained in Realgar is still a potentially toxic substance. Realgar and its compounds can cause adverse reactions or chronic arsenic poisoning when used chronically, in excess, and irregularly (Yi et al., 2018; Zheng et al., 2019). Some studies suggested that exposure to Realgar can lead to arsenic and various arsenical metabolites accumulation in the kidneys, which is a target organ for its metabolism and excretion (Wu et al., 2019). Thus, the metabolism of arsenic in Realgar-induced toxicity plays a leading role. Our previous work also revealed that Realgar can cause acute kidney injury and it is mainly related to the metabolic pathways (Zhang et al., 2021). Main recent studies concentrated on the determination of arsenic levels in blood, urine, and tissues (Wu et al., 2019; Yi et al., 2020). However, the metabolism changes of Realgar are still unclear.

Metabolomics is a systemic approach by applying modern advanced mass spectrometry techniques to identify and quantify mainly endogenous metabolites (around smaller than 1,000 Da) of complex systems. This consists of the holistic thinking of TCMs and metabolomics techniques are extremely suitable for evaluating the toxicity of traditional Chinese medicines and exploring the mechanisms (Wang et al., 2011). Recently, studies on the toxicological effects of Realgar or Realgar-containing medicines have been reported, and have mainly focused on hepatotoxicity (Zhang et al., 2017) and neurotoxicity (Zhang et al., 2019). However, less metabolic analysis of nephrotoxicity has been reported.

In this study, the nephrotoxic mechanism of Realgar was investigated by an untargeted metabolomic approach to analyze serum and kidney samples collected from control mice and mice treated with different doses of Realgar, which is expected to deepen the understanding of metabolic changes after exposure to Realgar.

## Materials and Methods

### Materials and Reagents

Realgar was provided and identified by the Shanghai Institute for Food and Drug Control (Shanghai, China) with 73.7% total arsenitc determined by Atomic Absorption Spectroscopy. Sodium carboxymethycellulose (CMC-Na) was purchased from Sinopharm (Beijiing, China). Mass spectrometry reagents including methanol, acetonitrile and formic acid were purchased from Sigma Chemical Co. (St. Louis, MO, United States).

### Animals and Treatment

**FIGURE 1:**
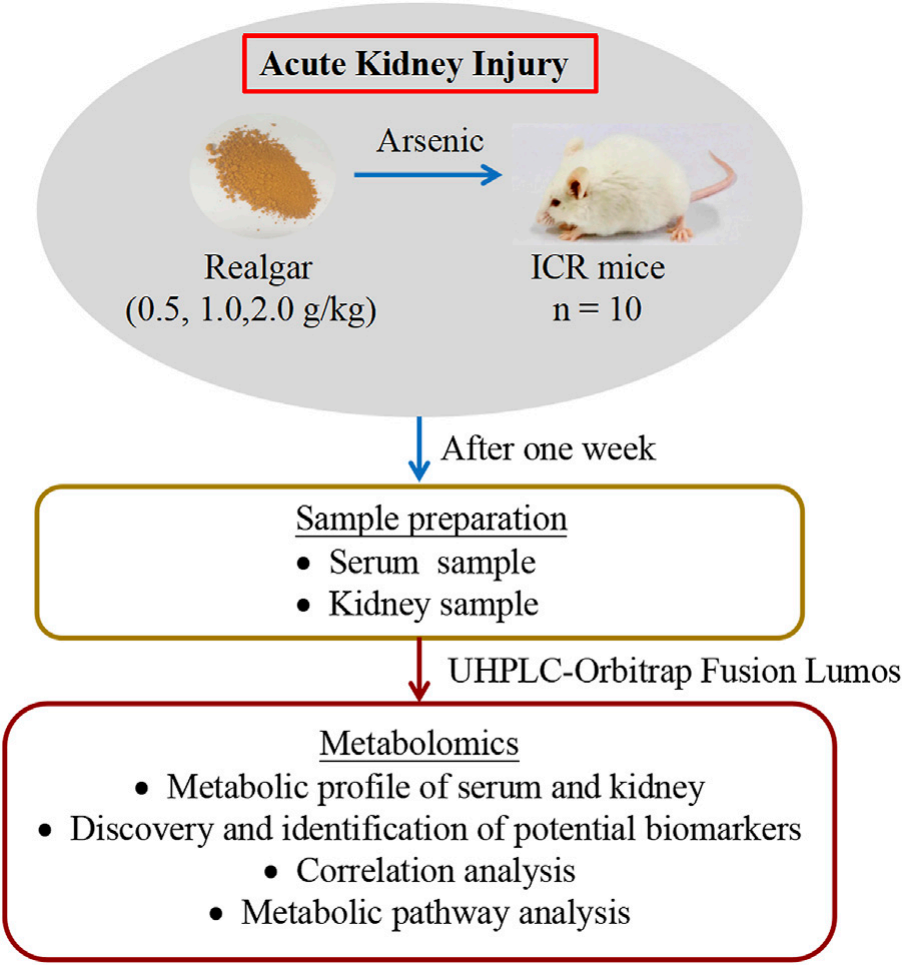
Experimental procedure of ICR mice exposed to Realgar.

Male 6-week-old ICR mice weighing about 18–20 g were provided by the Experimental Animal Center of Shanghai Institute for Food and Drug Control (SCXK 2018-0006, Shanghai, China). Mice were maintained at temperature (20–25°C) and 40–60% relative humidity for a 12-h light/dark cycle. They were fed freely water and diet, after 1 week of acclimatization, the mice were randomly divided into four groups for the treatment of 0.5% CMC-Na (Control group), Realgar suspended in 0.5% CMC-Na (0.5, 1.0, and 2.0 g/kg), respectively. The doses of Realar were referred to the basis of previously published literature (Miao et al., 2011; Xia et al., 2018). The detailed experiment procedure was shown in Figure 1. The body weight was measured every 2 days. After 1 week, blood samples were collected from the retro-orbital sinus of mice, and serum was collected after centrifugation. The mice were anesthetized, and the kidneys were collected immediately on ice and stored at −80°C for metabolomic analysis. The experimental protocol was performed according to the Animal experiment guidelines and approved by the animal Ethics Committee of Shanghai Institute for Food and Drug Control (IACUC-SIFDC20111).

### Sample Preparation

Serum samples were collected after following oral feeding Realgar for 30 min, then 50 μL of serum was mixed with 200 μL pre-cooled methanol and vortex mixed for 10 min. The mixture was then centrifuged at 14,000 rpm for 15 min at 4°C, and the supernatant is dried with nitrogen and reconstituted with 20% methanol. The supernatant was centrifuged at 14,000 rpm for 15 min at 4°C and used to analyze by LC-MS. A mixture of equal quantities was extracted from all samples and used as quality control (QC) sample for the LC-MS analysis.

The kidney tissues were dried by lyophilization and ground into powder. The powder (10 mg) was homogenized with pre-cooled 80% methanol (300 µL), mixed by vortexing, and subjected to sonication for 10 min on iced water (×3). The homogenate was then incubated at −20°C for 30 min and centrifuged at 14,000 rpm for 20 min at 4°C. The supernatant is dried with nitrogen and reconstituted with 80% methanol and centrifuged at 14,000 rpm at 4°C for 15 min. The supernatant was added to the sample bottle for detection. A mixture of equal quantities was extracted from all samples and used as QC sample for the LC-MS analysis.

### LC-MS Data Analysis

Kidney metabolic profiling adopted an UltiMate 3,000 ultra-high-performance LC system (Thermo Fisher Scientific) ACQUITY UPLC^®^ BEH C_18_ column (2.1 × 100 mm, 1.7 µm; Waters Corp.), coupled with Orbitrap Fusion Lumos high-resolution mass spectrometer (Thermo Fisher Scientific, USA). The mobile phase consisted of solvent A (0.1% formic acid in water) and solvent B (acetonitrile) with running a gradient elution: 5% phase B for 4 min; 5–70% phase B for 7 min, 70–95% phase B for 15 min, 95% for 17 min, 95–5% phase B for 20 min at the flow rate of 0.2 ml/min. The injection volume was 5 μL. The MS signal acquisition was performed in positive and negative ion scanning modes respectively. One primary mass spectrometry scan resolution is 15,000 and secondary mass spectrometry scans resolution is 6,000, and both the first and secondary scanning ranges were 105 m/z—1,050 m/z. A QC sample was acquired for every six analytical samples to evaluate the stability of the analytical system and assess the reliability of the results.

### Data Analysis

All data were presented as the mean ± SEM. The comparisons between two groups were performed with an unpaired two-tailed Student’s t-test using SPSS 23.0. The comparisons between several groups were performed with a one-way analysis of variance (ANOVA) using SPSS 23.0. Value of *p* < 0.05 was considered significant differences. The mass spectrometry-generated metabolomics data is in RAW file format. The quantitative analysis of the compound was conducted using Compound Discoverer Software 3.2 (Thermo Fisher Scientific, USA). The data was subject to principle components analysis (PCA) and orthogonal partial least squares discriminant analysis (OPLS-DA) using SIMCA-P version 14.1 software. The metabolites with VIP >1 and *p* < 0.05 were considered as potential biomarkers, and the online databases HMDB (http://www.hmdb.ca) were used to identify the compounds by matching the mass spectrum. Pathway enrichment was acquired according to the MetaboAnalyst 5.0 (http://www.metaboanalyst.ca/) and the Kyoto Encyclopedia of Genes and Genomes (KEGG, https://www.kegg.jp/) database.

## Result

### Weight Change and Kidney Weight Index

The body mass and kidney index of mice are shown in Table 1. Realgar at the dose of 2.0 g/kg had an effect on the decreased body weight (*p* < 0.05) and increased kidney weight index (*p* > 0.05).

**TABLE 1:** Effect of Realgar on body weight and kidney index of control and experimental animals.

Group	Body weight		Kidney weight (g)	Kidney weight index (%)
	Initial (g)	Final (g)		
Control	21.06 ± 0.71	21.87 ± 1.60	0.36 ± 0.05	1.63 ± 0.28
0.5 g/kg Realgar	21.22 ± 0.92	20.89 ± 0.94	0.35 ± 0.05	1.66 ± 0.21
1.0 g/kg Realgar	21.47 ± 0.58	21.7 ± 0.92	0.36 ± 0.04	1.68 ± 0.19
2.0 g/kg Realgar	21.33 ± 0.69	20.00 ± 1.12^*^	0.35 ± 0.05	1.74 ± 0.25

Values were measured as mean ± SEM (*n* = 10). Compared with the control group, *p < 0.05.

### Realgar Altered Serum and Kidney Metabolic Profile

**FIGURE 2:**
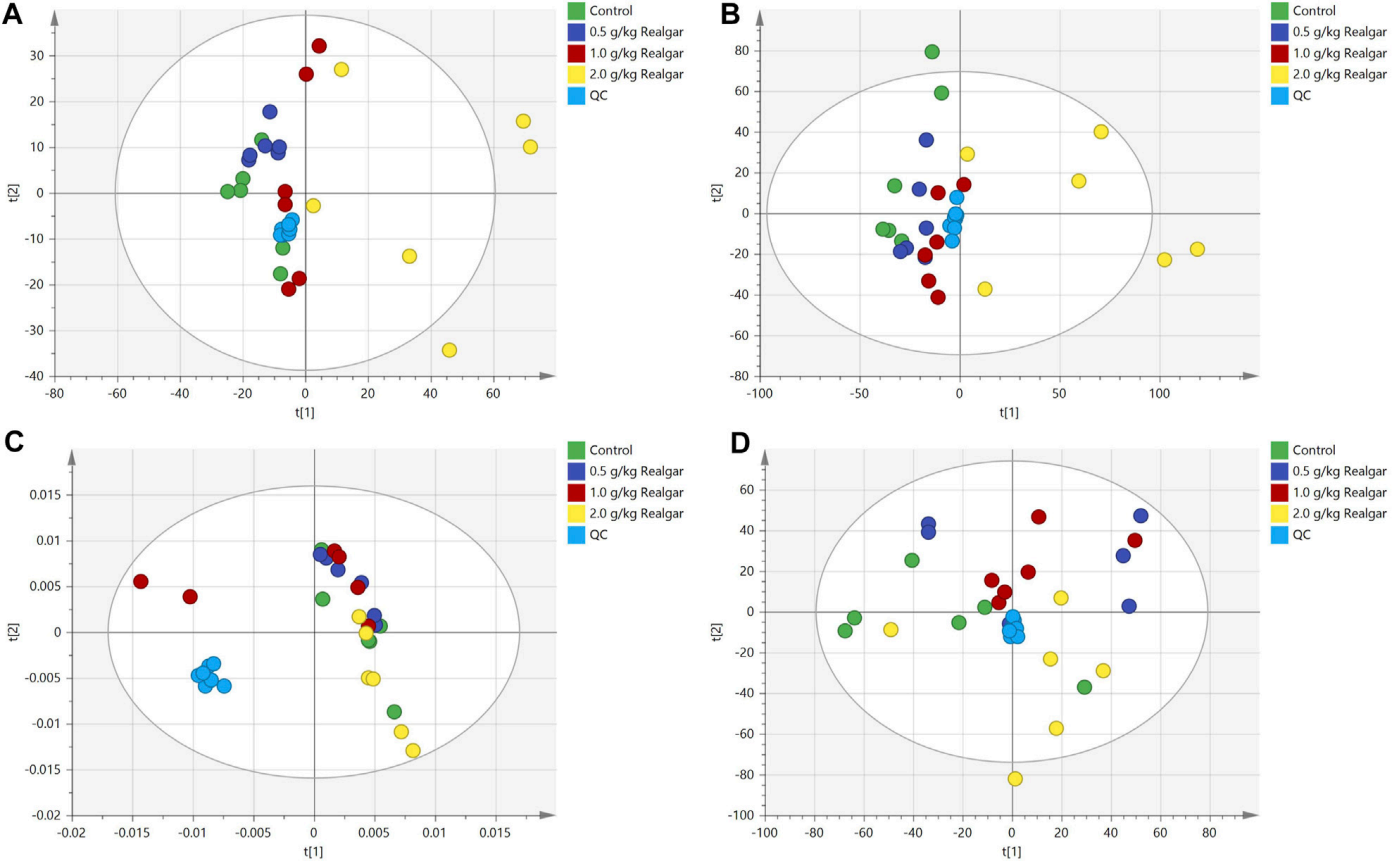
Scoring plots of PCA analysis [**(A and B)** of the positive and negative ion modes of serum samples, respectively; **(C and D)** of the positive and negative ion modes of the kidney samples, respectively] models with the statistical parameters as follows: **(A)** R2X = 0.495, Q2 = 0.138; **(B)** R2X = 0.388, R2Y = 0.841, Q2 = 0.530; **(C)** R2X = 0.525, Q2 = 0.207; **(D)** R2X = 0.391, R2Y = 0.867, Q2 = 0.552.

The PCA score plots shown good system stability of the analyses since the QC samples were tightly clustered in the serum and kidney samples (Figure 2), and all of the samples were within the confidence interval and the PCA showed no abnormal samples. Two principal components were obtained in positive and negative modes respectively. A slight separation for both serum and kidney samples was presented between the Control group and the Realgar groups (especially 2.0 g/kg Realgar group) in the PCA score plot, which may be due to large differences between samples.

### Discovery and Identification of Potential Biomarkers

**FIGURE 3:**
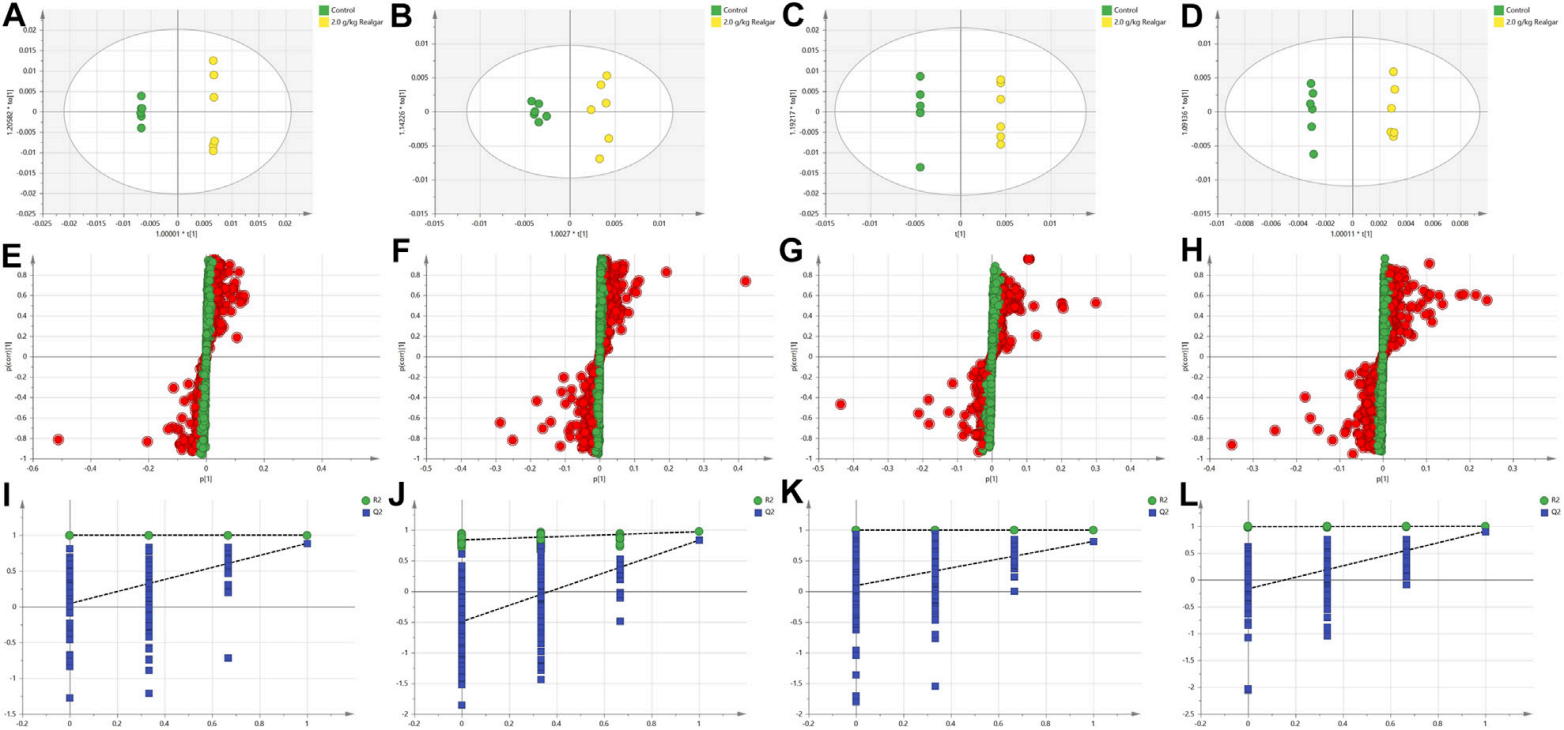
OPLS-DA analysis [**(A and B)** of the positive and negative ion modes of serum samples, respectively; **(C and D)** of the positive and negative ion modes of the kidney samples, respectively], Permutation test results [**(E and F)** of the positive and negative ion modes of serum samples, respectively; **(G and H)** of the positive and negative ion modes of the kidney samples, respectively], and S-plot (I and J of the positive and negative ion modes of serum samples, respectively; **(K and L)** of the positive and negative ion modes of the kidney samples, respectively). The models with the statistical parameters as follows: **(A)** R2X = 0.768, R2Y = 1, Q2 = 0.884; **(B)** R2X = 0.434, R2Y = 0.973, Q2 = 0.834; **(C)** R2X = 0.897, R2Y = 1, Q2 = 0.814; **(D)** R2X = 0.654, R2Y = 0.999, Q2 = 0.904; **(I)** R2 inter = 0.999, Q2 inter = 0.0432; **(J)** R2 inter = 0.828, Q2 inter = −0.494; **(K)** R2 inter = 0.1, Q2 inter = 0.0944; **(L)** R2 inter = 0.99, Q2 inter = −0.167.

The 2.0 g/kg dose group revealed a significant change of metabolites in comparison to the control group, so the OPLS-DA was used to monitor the changes of metabolites in the 2.0 g/kg dose group and the control group (Figure 3). R2X (cum) and Q2 (cum) parameters showed the model good fitting and prediction ability, and the separation between the control group and the 2.0 g/kg dose group was significant in the OPLS-DA score plot, indicating that the metabolite content of the 2.0 g/kg dose group of Realgar was different from that of the control group. Permutation tests with 200 iterations showed that the model was not over-fitted (Figure 3). As a visualization method, the S-plot was also used to select potential biomarkers (Figure 3). The importance of variables to classification was measured by the variable importance in the projection (VIP) value, and the variables were further screened according to VIP.

**FIGURE 4:**
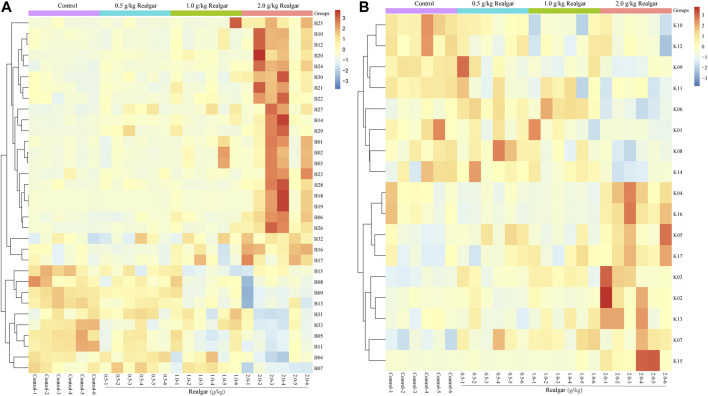
Heatmap of potential biomarkers of **(A)** serum and **(B)** kidney (*n* = 6). Rows represent the relative intensities of metabolites, where red represents high relative abundance metabolites and blue represents low relative abundance metabolites. Columns represent individual serum or kidney samples.

The potential biomarkers then were screened based on the strict criteria: 1) the variables with VIP value more than the one is an important screening parameter; 2) the *p* value less than 0.05 is considered as a significant difference in the candidate metabolite between the Control group and Realgar groups; 3) online databases HMDB (http://www.hmdb.ca) was searched to characterize differential metabolites by matching the mass spectrum; and 4) an error of less than 5 ppm. Following the above criteria, then thirty-three metabolites from serum samples and twenty-three from kidney samples were identified considered as potential biomarkers (Supplementary Tables 1, 2). In order to find the metabolites most related to Realgar-acute kidney injury, Spearman’s rank correlation analysis was performed using SPSS 23.0 software between the relative intensity (total area normalization) of metabolites and Realgar dose. Thirty-three metabolites from serum samples and seventeen from kidney samples were screened (Tables 2, 3 and Supplementary Tables 3, 4). To further understand metabolic differences among the Control group and treatment groups at different doses of Realgar, the metabolites were visualized in a clustering heatmap (Figure 4). The trend of metabolites in the Control group and 2.0 g/kg Realgar group could be easily observed from the heatmap in the mice serum, and similar results were observed in kidney tissues.

**TABLE 2:** Thirty-three potential biomarkers in serum samples of mice.

No	Compound name	Ion m/z	tR/min	Adduct	ESI mode
B01	Ornithine	133.09679	1.151	M + H	+
B02	L-Citrulline	176.10292	1.209	M + H	+
B03	L-Histidine	156.07591	1.185	M + H	+
B04	L-Arginine	175.11879	1.308	M + H	+
B05	(±)-2,2′-Iminobispropanoic acid	162.07614	1.632	M + H	+
B06	D-Alanyl-D-alanine	161.09172	1.626	M + H	+
B07	Acetyl-β-methylcholine	160.13289	1.634	M + H	+
B08	L-(-)-Methionine	150.05814	1.641	M + H	+
B09	Cytosine	112.05037	1.638	M + H	+
B10	Imidazolelactic acid	157.06061	1.657	M + H	+
B11	3-Methylglutaconic acid	145.04926	1.638	M + H	+
B12	Urocanic acid	139.04988	1.639	M + H	+
B13	Proline betaine	144.10184	1.633	M + H	+
B14	Bilirubin glucuronide	761.30304	9.135	M + H	+
B15	Taurine	124.00729	1.297	M-H	-
B16	Rhamnose	163.06111	1.421	M-H	-
B17	D-Xylulose	149.04559	1.417	M-H	-
B18	Aminoadipic acid	160.06155	1.43	M-H	-
B19	D-Proline	114.0559	1.441	M-H	-
B20	Ophthalmic acid	288.12036	1.566	M-H	-
B21	Pyroglutamic acid	128.03526	1.627	M-H	-
B22	5-Oxoproline	128.035	2.25	M-H	-
B23	Glutaric acid	131.03458	4.891	M-H	-
B24	Indole-3-carboxaldehyde	144.04536	8.675	M-H	-
B25	Taurocholic acid	514.28357	8.837	M-H	-
B26	Sulfolithocholylglycine	512.26813	9.012	M-H	-
B27	Glycochenodeoxycholate-3-sulfate	528.26221	9.067	M-H	-
B28	Taurochenodesoxycholic acid	498.28876	9.102	M-H	-
B29	7-Sulfocholic acid	487.23755	9.166	M-H	-
B30	5b-Cyprinol sulfate	531.29938	9.105	M-H	-
B31	LysoPE (0:0/20:3 (11Z,14Z,17Z))	502.29297	10.829	M-H	-
B32	LysoPE (18:1 (9Z)/0:0)	478.29337	11.188	M-H	-
B33	LysoPE (16:0/0:0)	452.27777	11.957	M-H	-

**TABLE 3:** Seventeen potential biomarkers in kidney samples of mice.

No	Compound name	Ion m/z	tR/min	Adduct	ESI mode
K01	Taurine	126.02162	1.673	M + H	+
K02	3-Methylcrotonylglycine	158.08072	1.939	M + H	+
K03	Acetylcholine	148.09688	1.924	M + H	+
K04	LysoPE (0:0/20:5 (5Z,8Z,11Z,14Z,17Z))	500.2767	11.074	M + H	+
K05	LysoPC(20:3 (5Z,8Z,11Z)/0:0)	546.35529	12.511	M + H	+
K06	Glycerol 3-phosphate	171.0062	1.32	M-H	-
K07	Galactitol	181.07175	1.274	M-H	-
K08	3-Sulfinoalanine	152.00211	1.377	M-H	-
K09	Homocysteinesulfinic acid	166.01773	1.45	M-H	-
K10	2-Oxoglutaramate	144.03000	1.555	M-H	-
K11	5-Acetamidovalerate	158.08205	1.559	M-H	-
K12	Inosine	267.07327	1.52	M-H	-
K13	5-Oxoproline	128.035	2.426	M-H	-
K14	Hexanoylglycine	126.09225	8.822	M-H	-
K15	Glycocholic acid	464.3017	9.378	M-H	-
K16	LysoPE (20:5 (5Z,8Z,11Z,14Z,17Z)/0:0)	498.2629	10.719	M-H	-
K17	LysoPE (20:3 (5Z,8Z,11Z)/0:0)	502.29373	12.095	M-H	-

### Correlation Analysis

**FIGURE 5:**
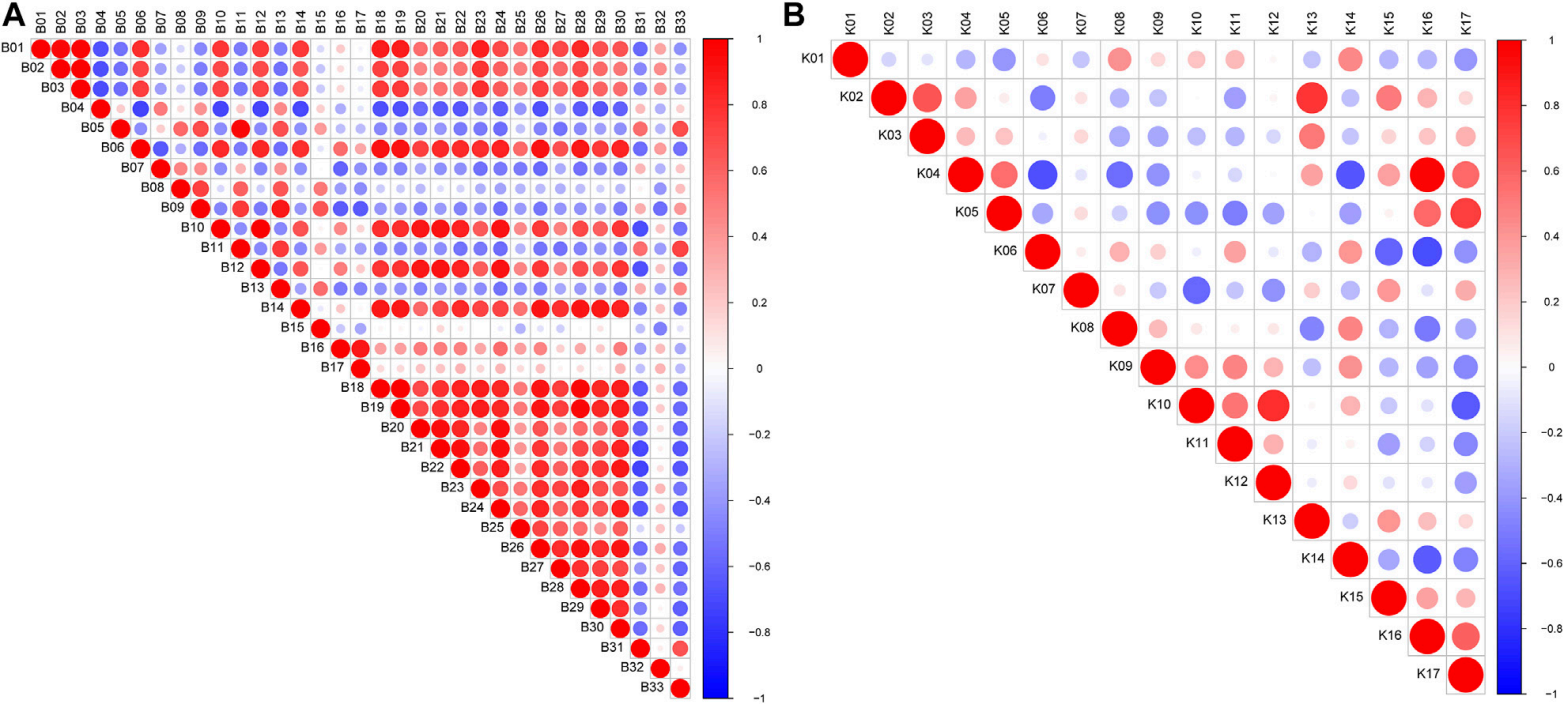
Pearson’s correlations of marker metabolites detected by LC/MS in mice **(A)** serum and **(B)** kidney. Red color indicates positive correlation between compounds and blue color indicates negative correlation between compounds; and the darker the color, the stronger the correlation.

To further understand the relationships between metabolites, Pearson’s correlation analysis was performed and shown in Figure 5. Most of the metabolites in the mice had a strong correlation, and they may have similar metabolic pathways and related biological effects. For example, metabolites between amino acid and bile acid components showed a high positive correlation in the serum samples, and there was a high positive correlation between the lipid components in the kidney samples.

### Metabolic Pathway Analysis

**FIGURE 6:**
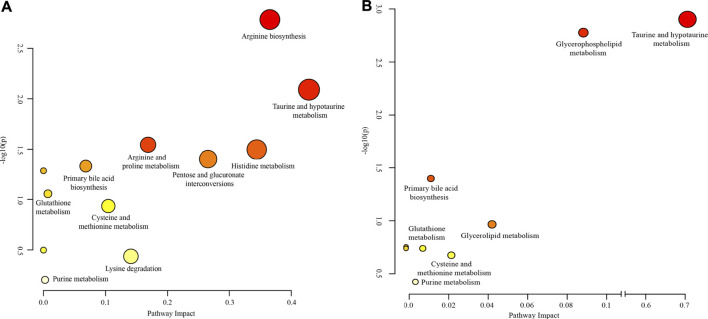
Weighted analysis of the metabolic pathways based on the potential biomarkers in **(A)** serum and **(B)** kidney samples.

To further clarify the possible metabolic pathways that were affected by Realgar, the metabolites in serum and kidney tissues were integrated and imported into Metaboanalyst 5.0 for pathway analysis, and the pathway was filtered by the parameter Impact >0. As shown in Figure 6 and Supplementary Tables 5, 6, ten metabolic pathways were highlighted from serum samples focusing on including Amino acid metabolism, Glutathione metabolism, and Purine metabolism, and seven metabolic pathways were highlighted from kidney samples focusing on including Amino acid metabolism, Lipid metabolism, Glutathione metabolism, and Purine metabolism.

## Discussion

The traditional toxicity evaluations showed that Realgar treatments elicited nephrotoxicity, as characterized by elevated blood urea nitrogen, creatinine levels in serum, and kidney histopathologic alterations. These have been confirmed in our previous studies, and Realgar at a high dose induced more severe acute kidney injury than exposure to a low dose (Zhang et al., 2021). In this study, a significant reduction in body weight were observed in the 2.0 g/kg Realgar group, which may be due to persistent non-remission of Realgar-acute kidney injury, increased toxin levels, and inadequate intake of ingested nutrients. Moreover, our previous study also found that metabolic disruption was observed in the kidney of mice exposed to Realgar for 1 week (Zhang et al., 2021). Therefore, a metabolomic approach was used to further investigate the mechanism of acute nephrotoxicity of Realgar-induced acute nephrotoxicity in mice.

**FIGURE 7:**
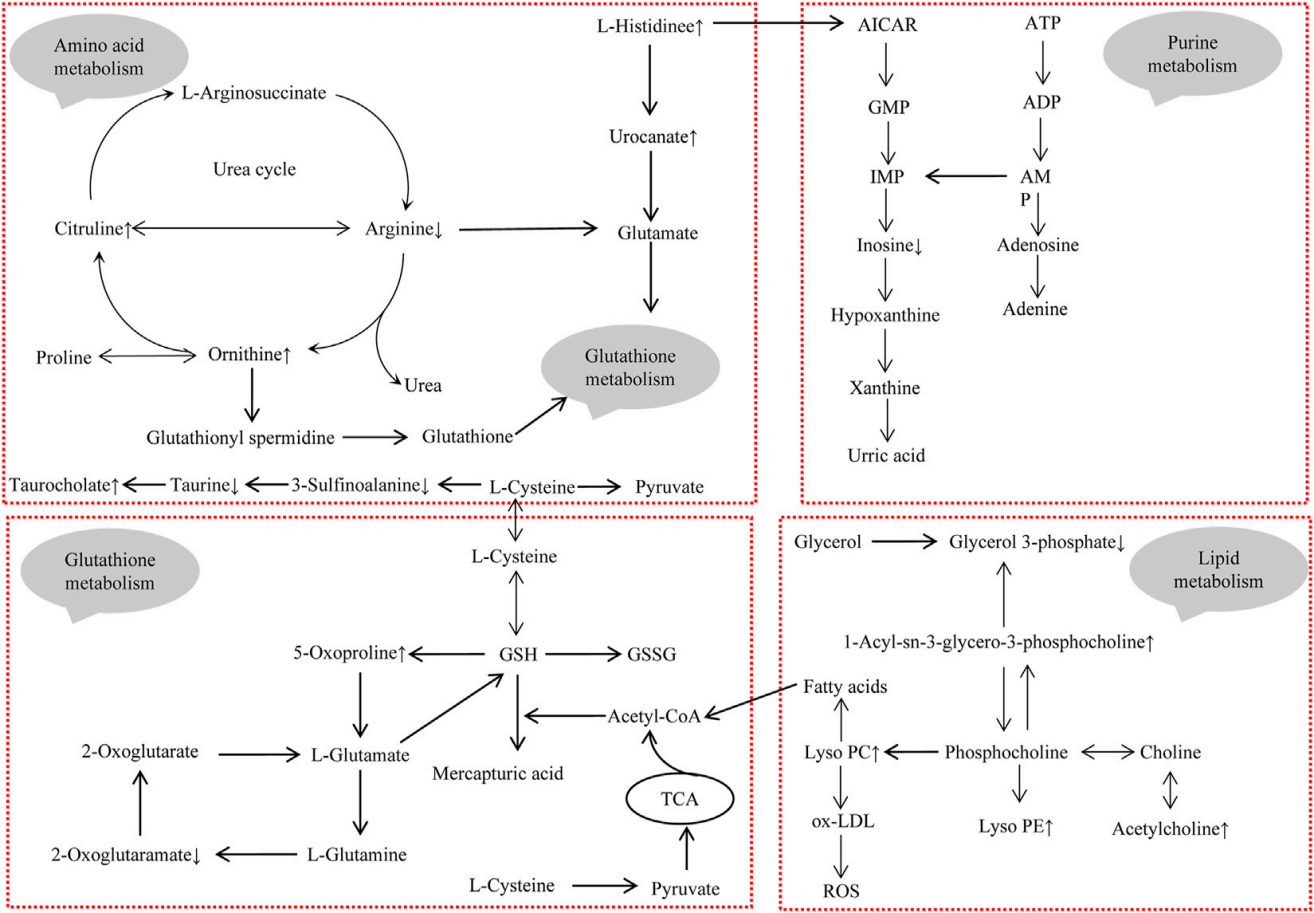
The integrated metabolic pathway related to Realgar-induced acute kidney injury. Upward arrows indicate elevated levels after exposure to Realgar, and conversely, downward arrows indicate decreased levels after exposure to Realgar.

High-resolution mass spectrometry combined with metabolomics was used to analyze metabolite alterations in serum and kidney of Realgar mice. Thirty-three endogenous metabolites in serum and seventeen endogenous metabolites in the kidney were selected as toxic biomarkers, which are mainly involved in amino acid metabolism, Lipid metabolism, Glutathione metabolism, and Purine metabolism. Amino acid metabolism, Glautathione metabolism, and Purine metabolism were the same metabolic pathways between serum and kidney, and Lipid metabolism might be partly attributed to oxidative stress caused by Realgar. Therefore, these four pathways were of great attention to us. The mechanism of toxicity was proposed by analyzing their formation and transformation in the pathway (Figure 7).

### Amino Acid Metabolism

Taurine has many different biological functions, bile acid binding, antioxidant, etc., which are important for organism health and disease. Taurine has been reported to have a protective effect against drug-induced nephrotoxicity (Aslanturk and Uzunhisarcikli, 2020). In Realgar-induced hepatotoxicity, the level of taurine was decreased (Huo et al., 2016). In this study, taurine levels were decreased in both serum and kidney of mice. Therefore, taurine may be an important potential marker associated with Realgar-induced nephrotoxicity.

Histidine, Citrulline, Ornithine were only detected in serum samples, and the disruption of amino acid metabolism was observed mainly in serum samples. Histidine is one of the 22 proteinogenic amino acids and can be catabolized to glutamate, which has antioxidant properties (Brosnan and Brosnan, 2020). In this study, Histidine and the catabolic product urocanic acid were increased, indicating that the oxidative response has been activated in mice. Notably, increased level of Histidine in the blood is also a major manifestation of congenital disorders of Histidine metabolism, including histidinemia, maple syrup urine disease, propionic acidemia, and tyrosinemia I (Moro et al., 2020). Ammonia accumulation occurs when amino acid metabolism is disturbed and the urea cycle converts ammonia to urea, which is excreted in the urine. Citrulline, Ornithine, and Arginine play a key role in the urea cycle (Morris, 2002). The increased levels of Citrulline and Ornithine in serum after exposure to Realgar implied an acceleration of the urea cycle. The decreased level of arginine was observed and may reduce the production of glutamate. In addition, increased serum levels of bile acids may be associated with deranged amino acid metabolism.

### Lipid Metabolism

Some reports have shown that Realgar can cause the disorders of lipids metabolism in rats and mice (Zhang et al., 2019; Zhang et al., 2017), Pearson’s correlation analysis from kidney samples also indicated that the perturbation of lipids metabolism may play an important role in the nephrotoxicity induced by Realgar. In this study, the increased levels of lysophosphatidylcholine (LPC) and lysophosphatidyl ethanolamine (LPE) were detected in kidney tissue aqueous extracts after Realgar treatment. PC, PE, LPC, and LPE are the main phospholipid components to constitute the cell membrane structure and can mutually transform (Fujita et al., 2014; Canning et al., 2016). LPC is an important fragment of ox-LDL, notably, ox-LDL is an indicator of lipid oxidation that induces ROS formation and causes oxidative damage to cells (Bao et al., 2018). Furthermore, LPC can directly increase Ca^2+^ concentration and induce ROS production to initially induce oxidative stress (Jung et al., 2017). Arsenic has also been shown to induce cellular autophagy, and PE is involved in the synthetic conversion of the key protein LC3 to LC3-II (Wei et al., 2019; You et al., 2019). However, the levels of LPE in serum were unstable, which may be due to serum metabolites come from multiple organs.

### Glutathione Metabolism

Glutathione (GSH), an important intracellular regulatory metabolite, is involved in several reaction systems, especially in defense against oxidative stress. In our study, pathway analysis showed that metabolites in serum and metabolites in the kidney simultaneously regulate glutathione metabolism. As shown in Figure 7, several metabolites (such as Ornithine, Arginine, 2-Oxoglutaramate, and 5-Oxoproline) are closely associated with glutathione metabolism, suggesting that Realgar induced glutathione metabolic processes in mice kidney tissue, which is consistent with the literature (Zhou et al., 2017). There may be an exchange of substances between serum metabolites and renal metabolites, with amino acids in serum providing the basic substances involved in glutathione metabolism, such as glutamate, which are metabolized by the kidneys and returned to serum. Furthermore, arsenic has been shown to cause toxicity mainly by binding to sulfhydryl-containing proteins (Mandal, 2017). The GSH molecule is characterized by an active sulfhydryl group, and also activates sulfhydryl (SH) enzyme-coenzyme activity, which can participate in heavy metal toxicity reactions (Chen et al., 2021).

### Purine Metabolism

Histidine metabolic disruption has been observed in this study, and its metabolite AICAR activates the purine metabolic process (Daignan-Fornier and Pinson, 2012). Most of the purine compounds are oxidized to uric acid, which is excreted in the urine, and the level of blood uric acid is closely related to kidney damage. However, in this study, changes in uric acid levels were not detected in either serum or kidney.

This study provided more comprehensive information about the toxicity of Realgar through testing both serum and renal metabolic profiling. However, limitations of this study need to be further addressed. Firstly, serum metabolites come from multiple organs, such as kidney, liver, intestine and so on. Therefore, this result is not specific for reflecting the nephrotoxicity of Realgar. This is one of the tasks that our research group is currently preparing to carry out. Secondly, some metabolites may be not associated with Realgar, but due to acute kidney injury. Acute kidney injury animal model will be established to screen out specific metabolites by comparing Realgar-induced acute kidney injury with other causes-induced acute kidney injury. In addition, we are also trying to find clinical samples of acute kidney injury patients.

## Conclusion

This work aimed to study Realgar-induced acute kidney injury through using a high-resolution mass spectrometry technology combined with a metabolomics approach. LC-MS analysis of mice serum and kidney highlighted some disturbances in the endogenous metabolite profile, which may be related to disturbances in the biochemical pathways of acute kidney injury induced by Realgar. In the study, amino acid metabolism is most affected by Realgar. Lipid metabolism, Glutathione metabolism, and Purine metabolism were also affected by Realgar. The high-resolution mass spectrometry combined with metabolomics can capture and explore the metabolic alterations induced by traditional Chinese medicines in toxicological effects and can be used to further investigate the toxicity mechanisms of traditional Chinese medicines.

## Data Availability

The original contributions presented in the study are included in the article/Supplementary Material, further inquiries can be directed to the corresponding author.
